# Dehydration and Suboptimal Sleep Aggravate Early Renal Impairment in Children: Longitudinal Findings from the PROC Study

**DOI:** 10.3390/nu16203472

**Published:** 2024-10-14

**Authors:** Menglong Li, Huidi Xiao, Nubiya Amaerjiang, Bipin Thapa, Wen Shu, Yeerlin Asihaer, Mengying Guan, Sten H. Vermund, Zhiyong Zou, Dayong Huang, Yifei Hu

**Affiliations:** 1 Department of Child, Adolescent Health and Maternal Care, School of Public Health, Capital Medical University, Beijing 100069, China; limenglong_ph@163.com (M.L.); xhd19988023@163.com (H.X.); 13693617970@163.com (N.A.); bipinthapa2050@gmail.com (B.T.); nmg_sw026@163.com (W.S.); yeerlin@mail.ccmu.edu.cn (Y.A.); gmying@mail.ccmu.edu.cn (M.G.); 2 Department of Pediatrics, School of Medicine, Yale University, New Haven, CT 06510, USA; sten.vermund@yale.edu; 3 Institute of Child and Adolescent Health, School of Public Health, Peking University, Beijing 100191, China; 4 Department of Hematology, Beijing Friendship Hospital, Capital Medical University, Beijing 100050, China; 5 UNESCO Chair on Global Health and Education, Peking University, Beijing 100191, China; 6 Beijing Key Laboratory of Environmental Toxicology, Capital Medical University, Beijing 100069, China

**Keywords:** hydration status, dehydration, sleep duration, kidney, renal function, children, pediatric population, cohort, longitudinal study, China

## Abstract

Background: While dehydration is associated with pediatric renal impairment, the regulation of hydration status can be affected by sleep. However, the interaction of hydration and sleep on kidney health remains unclear. Methods: We conducted a cohort study among 1914 healthy primary school children from October 2018 to November 2019 in Beijing, China. Four-wave urinary β_2_-microglobulin and microalbumin excretion were assayed to assess transient renal tubular and glomerular impairment, and specific gravity was measured to determine hydration status with contemporaneous assessment of sleep duration, other anthropometric, and lifestyle covariates. We used generalized linear mixed-effects models to assess longitudinal associations of sleep duration and hydration status with renal impairment. Results: We observed 1378 children with optimal sleep (9–<11 h/d, 72.0%), 472 with short sleep (<9 h/d), and 64 with long sleep (≥11 h/d, 3.3%). Over half (55.4%) of events determined across 6968 person-visits were transient dehydration, 19.4% were tubular, and 4.9% were glomerular impairment events. Taking optimal sleep + euhydration as the reference, the results of generalized linear mixed-effects models showed that children with long sleep + dehydration (odds ratio [OR]: 3.87 for tubular impairment [tubules] and 3.47 for glomerular impairment [glomerulus]), long sleep + euhydration (OR: 2.43 for tubules), optimal sleep + dehydration (OR: 2.35 for tubules and 3.00 for glomerulus), short sleep + dehydration (OR: 2.07 for tubules and 2.69 for glomerulus), or short sleep + euhydration (OR: 1.29 for tubules) were more likely to present transient renal impairment, adjusting for sex, age, body mass index z-score, systolic blood pressure z-score, screen time, physical activity, and Mediterranean diet adherence. Conclusions: Dehydration and suboptimal sleep aggravate transient renal impairment in children, suggesting its role in maintaining pediatric kidney health.

## 1. Introduction

Modern lifestyles, economic development, and preventive medicine have led to a decreased incidence of common pediatric infectious diseases and nutritional deficiencies, with a relative increase in the incidence of chronic threats, including kidney disease [1,2]. Childhood is a critical period both for the maturation of renal function and the formation of a healthy lifestyle [3,4]. Kidneys need to accommodate rapid growth in childhood and respond to physiological and environmental stresses. Transient impairment caused by responding to adverse stimuli is common even in individuals with normal renal function. Renal tubular and glomerular impairment result in elevated urinary protein excretion [3]. Urinary β_2_-microglobulin (β_2_-MG) and microalbumin (MA) are biomarkers indicating early tubular and glomerular impairment, respectively [5,6]. Repetitive or excessive transient impairment stresses the kidney’s compensatory capacity with a potential for later development of chronic kidney disease (CKD).

Sleep is essential for the neurobehavioral development of children and sleep disturbances have been associated with various health outcomes, including obesity, urolithiasis, albuminuria, renal function, and CKD [7,8,9]. In China, the average sleep duration of children aged 6–17 is 8.5 h, falling short of recommended guidelines [10]. High academic pressure may contribute both to sleep disturbances and altered water balance in the body [11,12]. Adequate hydration is crucial for optimal physiological functioning and organ health. Intensive academic schedules and short inter-curriculum breaks during the school days may lead students to drink less and reduce the micturition frequency [12]. Consequently, dehydration is common in children due to such behavioral and physiological reasons, and we have reported previously that dehydration is associated with transiently impaired renal function [13,14].

Dehydration can be associated with decreased blood volume, increased plasma sodium concentration/plasma osmolality, activation of the renin–angiotensin–aldosterone system, and release of vasopressin, contributing to renal inflammatory responses [15]. The release of vasopressin and regulation of hydration status is also affected by sleep and circadian rhythms [16]. Both short or long sleep durations are associated with dehydration compared to optimal sleep duration [17], and studies further suggest a dyadic disruption of homeostatic systems linked to sleep and hydration [7,8,9,17]. However, previous studies mostly reported independent associations regarding sleep and hydration with renal impairment, and the interaction between sleep and hydration on transient renal impairment has not been studied, particularly in a general pediatric population.

In this longitudinal study, we investigated transient renal tubular and glomerular impairment in an ongoing child cohort (PROC) study. We assessed associations and interactions of sleep duration and hydration status with transient renal impairment among general elementary school-aged children.

## 2. Materials and Methods

### 2.1. Study Population and Design

We enrolled 1914 children aged 6–8 years from the child cohort (PROC) study established in 2018 in Beijing, China. The PROC study (register number in Chinese Clinical Trial Registry: ChiCTR2100044027) used a community-based census-like design and recruited grade-one students from six non-boarding primary schools in urban Beijing and studied them longitudinally (detailed elsewhere [18]). In this study, four waves of urine assays were conducted: baseline survey from October to November 2018 and three follow-up visits within a one-week span in November 2019 (detailed elsewhere [14,19]). There were 1914 person-visits in wave one, 1746 person-visits in wave two, 1651 person-visits in wave three, and 1657 person-visits in wave four, for a total of 6968 person-visits.

### 2.2. Urine Assays and Outcomes

Baseline and follow-up urine sample collection and test procedures are detailed elsewhere [14,19]. In brief, fasting urine assays in wave one were conducted on any weekday, 24 h urine assays in wave two were conducted on Monday, and fasting urine assays in wave three were conducted on Wednesday and for wave four on Friday. Specific gravity (SG) was used to determine children’s hydration status, and β_2_-MG and MA were used to determine renal impairment. Dehydration was defined as SG ≥ 1.020, while SG < 1.020 indicated euhydration. Transient tubular impairment was defined as β_2_-MG > 0.2 mg/L and glomerular impairment as MA ≥ 20 mg/L in urine samples.

### 2.3. Exposure and Covariates

The principal independent variables were based on our hypothesis, namely hydration status and sleep duration of the children reported by their parents via the Children’s Sleep Habits Questionnaire (CSHQ). The average daily sleep duration was calculated as (5 × weekday sleep duration + 2 × weekend sleep duration)/7. Short sleep for children was defined as sleep duration <9 h/day (h/d), optimal sleep was 9–<11 h/d, and long sleep was ≥11 h/d according to the Canadian 24-Hour Movement Guidelines for Children and Youth [20]. Via examinations and questionnaires, we assessed key anthropometric and lifestyle covariates including height, weight, body mass index (BMI), systolic blood pressure (SBP), and diastolic blood pressure (DBP) as detailed elsewhere [14,19]. Z-scores of height, weight, and BMI were calculated using 2007 WHO standards. Standardized SBP was calculated for each age and sex group. Parents reported lifestyle information using self-administered questionnaires; this included estimates of computer/cell phone screen time, physical activity (PA) time (Children’s Leisure Activities Study Survey Chinese version [CLASS-C]), and the adherence to a Mediterranean Diet (the 16-item Mediterranean Diet Quality Index in children and adolescents [KIDMED] with a score range of 0–12). Activity time was reported daily for one week and was calculated as a daily average in hours over a period of seven days. Long screen time was defined as screen time ≥2 h/d. Insufficient physical activity was defined as <1 h/d. Poor Mediterranean diet adherence was defined as 0–3 scores [21].

### 2.4. Statistical Analysis

Descriptive statistics were presented by sleep duration categories. Continuous covariates such as z-scores of height, weight, and BMI and blood pressure were presented as mean ± standard deviation (SD), whereas categorical covariates like sex and lifestyle-related variables were presented as counts and percentages. Analysis of variance (ANOVA), Chi-square, and Fisher’s exact tests were used to assess statistical differences in variables between sleep duration groups. Trend Chi-square tests were performed to determine the trends in the prevalence of transient renal impairment across different study waves. Generalized linear mixed-effects models were developed to determine the longitudinal associations and odds ratio (OR) with a 95% confidence interval (CI) of the direct effect and interaction of sleep duration and hydration status on renal impairment. The weekdays of the urine assays were included as random effects. Multiple imputations were conducted for variables with missing values [22] with 50 complete imputed datasets obtained for further analysis. A two-tailed *p* value < 0.05 was used to determine statistical significance. We used Statistical Analysis System V.9.4 (SAS Institute Inc., Cary, NC, USA) for analyses.

## 3. Results

**Figure 1:**
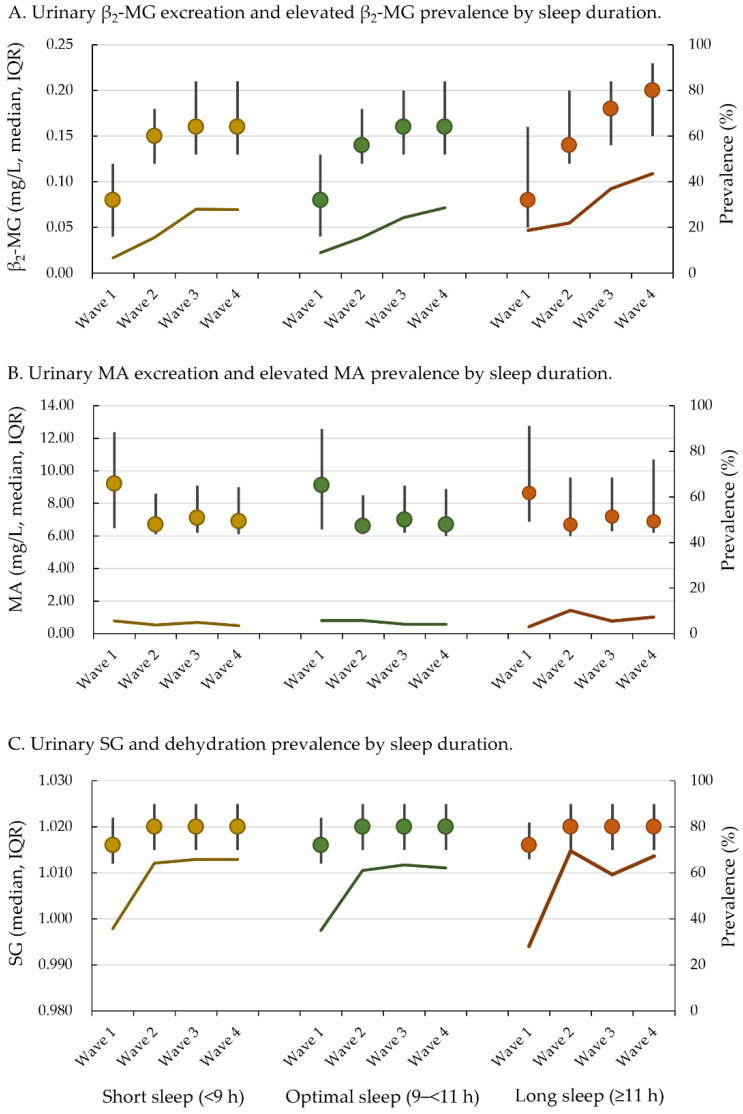
Median and prevalence of urinary indicators across four waves of this study by sleep duration. β_2_-MG: β_2_-microglobulin; MA: microalbumin; SG: specific gravity; IQR: interquartile range.

From October 2018 to November 2019, 1914 participants (956 boys and 958 girls) with a mean age of 6.6 ± 0.3 years at baseline were enrolled in this study with an eventual total of 6968 person-visits. The prevalence of short sleep duration (<9 h/d) was 24.7% (472 children with 1724 person-visits), optimal sleep duration (9–<11 h/d) was 72.0% (1378 children with 5012 person-visits), and long sleep duration (≥11 h/d) was 3.3% (64 children with 232 person-visits), with no sex differences (Table 1). Across the four waves, the prevalence of dehydration at baseline wave 1 was 35.0% (671/1914), wave 2 was 62.1% (1085/1746), wave 3 was 63.9% (1055/1651), and wave 4 was 63.3% (1049/1657) with an overall rate of 55.4% of the total person-visits. We observed an increasing trend in renal tubular impairment events (8.8%, 15.9%, 25.7%, and 29.0% from waves 1 to 4, respectively; *Z* = 16.9, *p* < 0.001) with an overall rate of 19.4% for the total person-visits. A decreasing trend of glomerular impairment events (5.6%, 5.5%, 4.4%, and 4.1% from waves 1 to 4, respectively; *Z* = −2.4, *p* = 0.02) was seen with an overall rate of 4.9% for the total person-visits in this general pediatric population. Prevalence of urinary indicators across four waves by sleep duration are presented in Figure 1.

Insufficient physical activity and poor Mediterranean diet adherence were more prevalent in children having short sleep duration. There were no significant differences in sex, age, height z-score, weight z-score, BMI z-score, SBP, and DBP between the three sleep duration groups (Table 1).

### 3.1. Sleep Duration and Renal Impairment in Children

We observed statistically significant longitudinal associations between sleep duration and transient renal tubular impairment, with children who had longer sleep duration being more likely to have transient renal tubular impairment compared to those with optimal sleep duration in unadjusted model 1 (OR = 1.84, 95% CI: 1.37, 2.48), model 2 adjusted for sex, age, BMI z-score (OR = 1.89, 95% CI: 1.40, 2.55), and model 3 adjusted for sex, age, BMI z-score, SBP z-score, screen time, PA level, and Mediterranean diet adherence (OR = 1.88, 95% CI: 1.39, 2.54). No significant longitudinal association was found between short sleep duration and renal tubular or glomerular impairment, nor was there any significant longitudinal association between long sleep duration and renal glomerular impairment (Table 2).

### 3.2. Hydration Status and Renal Impairment in Children

Dehydration status was significantly and positively associated with both renal tubular and glomerular impairment in models 1–3. Children with dehydration status were more likely to have a transient renal tubular impairment (OR = 2.10, 95% CI: 1.83, 2.40) and renal glomerular impairment (OR = 2.97, 95% CI: 2.27, 3.88), compared to those with euhydration status (model 3, Table 2).

### 3.3. Impact of Suboptimal Sleep and Dehydration on Pediatric Renal Impairment

Multivariable analysis showed similar results when including sleep duration and hydration status in one model. Children with long sleep duration (OR = 1.90, 95% CI: 1.40, 2.57) and dehydration (OR = 2.10, 95% CI: 1.83, 2.40) were more likely to have renal tubular impairment compared to those with optimal sleep duration and euhydration status (reference population). Similarly, children who were dehydrated were more likely to have a renal glomerular impairment (OR = 2.97, 95% CI: 2.27, 3.88) compared to their counterparts (model 3, Table 3).

### 3.4. Interaction of Sleep and Hydration on Pediatric Renal Impairment

**Figure 2:**
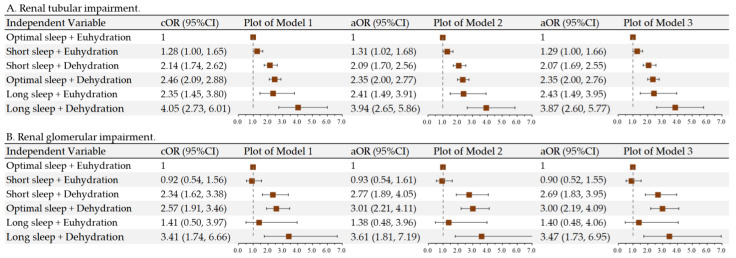
Longitudinal associations between the interaction of sleep duration and hydration status with renal tubular and glomerular impairment among children aged 6–9 years in Beijing, China. Model 1: unadjusted; model 2: adjusted for sex, age, and body mass index z-score; model 3: model 2 further adjusted for systolic blood pressure z-score, screen time, physical activity level, and Mediterranean diet adherence. cOR: crude odds ratio; aOR: adjusted odds ratio; CI: confidence interval. All models included one random effect: the weekday of the urine assay.

To examine the interaction between sleep duration and hydration status, we grouped them accordingly to assess their associations with transient renal impairments. Children with short sleep and euhydration (OR = 1.29, 95% CI: 1.00, 1.66), short sleep and dehydration (OR = 2.07, 95% CI: 1.69, 2.55), optimal sleep and dehydration (OR = 2.35, 95% CI: 2.00, 2.76), long sleep and euhydration (OR = 2.43, 95% CI: 1.49, 3.95), and long sleep and dehydration (OR = 3.87, 95% CI: 2.60, 5.77) were more likely to have renal tubular impairment compared to those having optimal sleep duration and euhydration status. Children with short sleep and dehydration (OR = 2.69, 95% CI: 1.83, 3.95), optimal sleep and dehydration (OR = 3.00, 95% CI: 2.19, 4.09), and long sleep and dehydration (OR = 3.47, 95% CI: 1.73, 6.95) were more likely to have transient renal glomerular impairment compared with those with optimal sleep duration and euhydration status. Sex, age, BMI z-score, SBP z-score, screen time, PA level, and Mediterranean diet adherence were adjusted in the models but were not highly influential (model 3, Figure 2).

### 3.5. Supporting Analyses

We further performed a stratified analysis to assess the role of hydration in renal impairment among children with different sleep durations. There was a trend, but no significant protective effect, of euhydration on renal impairment among children with a long sleep duration (OR = 0.61, 95% CI: 0.33, 1.13 for tubules; OR = 0.44, 95% CI: 0.09, 2.17 for glomerulus). Significant protective effects of euhydration on renal impairment were found among children with an optimal sleep duration (OR = 0.43, 95% CI:0.36, 0.50 for tubules; OR = 0.33, 95% CI: 0.24, 0.46 for glomerulus) and short sleep duration (OR = 0.62, 95% CI: 0.47, 0.81 for tubules; OR = 0.31, 95% CI: 0.17, 0.55 for glomerulus), after adjusting for sex, age and BMI z-score (Table 4).

## 4. Discussion

This study found that children with suboptimal sleep (<9 h/d or ≥11 h/d) or with dehydration were more likely to have transient renal impairment, especially tubular impairment in the general pediatric population of 6–9 years, after controlling the key anthropometric and lifestyle covariates. Moreover, we observed significant “dose-response” effects of sleep duration and hydration status on early renal impairment. Taking optimal sleep and euhydration status as a reference, children with short sleep and euhydration were 1.29-times more likely to have renal tubular impairment; children with short sleep and dehydration were 2.07- and 2.69-times more likely to have a renal tubular impairment and renal glomerular impairment, respectively; children with optimal sleep and dehydration were 2.35- and 3.00-times more likely to have renal tubular impairment and renal glomerular impairment, respectively; children with long sleep and euhydration were 2.43-times more likely to have renal tubular impairment; children with long sleep and dehydration were 3.87- and 3.47-times more likely to have renal tubular impairment and renal glomerular impairment, respectively. These findings suggest the necessity of healthy sleep and hydration patterns among elementary school children in urban China who are under considerable social stress towards high school performance. Along with optimizing sleep duration and maintenance of adequate hydration, we suggest regular monitoring of renal function via urine for the protection of kidney health in the general pediatric population.

We observed an increasing trend in the prevalence of transient renal tubular impairment across the four waves of measurements over the course of one year among primary school students. Tubular impairment was defined as β_2_-MG > 0.2 mg/L; therefore, the observed trend suggested two patterns for excreting more β_2_-MG, one with increasing enrollment years and the other with cumulative school days. β_2_-MG is a protein with a low molecular weight of 11.8 kilodaltons (kDa), which is freely filtered by the glomerulus and is completely hydrolyzed and reabsorbed in the renal tubules [5]. In the urine, β_2_-MG is virtually undetectable under normal physiological conditions, and an elevated concentration implies proximal tubular dysfunction [5]. We term this as “transient” since, to our knowledge, most our subjects did not have a diagnosed renal disease during the study time period; the children are still within the cohort and will be assessed for several more years. Our results are compatible with a hypothesis that stress on renal tubules increases during school days, which might be related to sitting position, prolonged sedentary periods, and excessive academic pressure that may not encourage rehydration breaks.

In contrast, a decreasing trend was observed in the prevalence of transient renal glomerular impairment across waves. MA, a protein with a molecular weight of 67 kDa, is recognized as a diagnostic indicator for early renal glomerular impairment [6]. Increased MA excretion is more common in the presence of glomerular podocyte injury, intraglomerular hypertension and hemodynamic dysfunction, and increased permeability of the filtration barrier due to stress or exercise [23]. We hypothesized that this decreasing trend in the prevalence of glomerulus impairment was associated with corresponding less PA in higher grades or on weekdays, as partially supported in the previous study [14]. 

In addition, our previous study found a stable occurrence of renal glomerular impairment among children with dehydration and a decreasing trend among well-hydrated children [13], which may contribute to the overall decreasing trend. Limited research has examined early impairment across various parts of the kidney, with most studies focused on the kidney function of pediatric patients. Our findings suggest early, transient renal tubular and glomerular impairment in this general pediatric population, with a prevalence of 19.4% and 4.9%, respectively.

The prevalence of abnormal sleep duration was 28% in our study (24.7% as short sleep duration and 3.3% as long sleep duration). The prevalence of long sleep duration in our study is higher than that of another Chinese national survey (0.1%) [24] and a cross-sectional study in Shanghai (0.69%) [25]. Meanwhile, the prevalence of short sleep duration is lower than that of the Chinese national survey (36.2%) [24] and between that of 64.5% on weekdays and 19.5% on weekends in an online survey in Singapore [26]. The variation in sleep duration prevalence may be attributed to differences in survey methods for assessing sleep duration, participant age ranges, and the definition of subgroups. However, the large sample size and validated CHSQ questionnaires used in our study ensured a good representation of the prevalence of abnormal sleep duration among grade-one primary school students. We found that insufficient PA and poor Mediterranean diet adherence were more prevalent among children with a short sleep duration, which was consistent with other studies [27,28,29,30,31,32]. We observed significant binary and longitudinal associations between long sleep duration and renal tubular impairment. Our findings in this healthy population of elementary school children are unique in the literature, we believe, and provide additional evidence to support consistent findings from several adult studies, that a long sleep duration is associated with a decline in kidney function [8,9,33,34]. The mechanisms underlying such association remain unclear with possible attributions including genetically driven alterations in the kidney phenotype, sleep disorders, circadian rhythm disorders, and immune system dysfunction [8,9,33,34]. Our results, which demonstrated a non-significant association between long sleep duration and transient glomerular impairment, suggest that the decline in tubular function may precede glomerular dysfunction. This highlights the necessity for early assessment of parts of the kidney in the pediatric population to maximize the prevention of kidney disease and underscore the importance of promoting change in modifiable factors, including health lifestyles, to prevent kidney disease.

The role of sufficient sleep or PA and a healthy diet for preventing non-communicable diseases are well-recognized; however, water intake or hydration status as one of the most important components of healthy lifestyles has been largely disregarded [15]. Our findings in the current study and previous studies showed that dehydration was significantly associated with renal tubular and glomerular impairment [13,14] and liver disease [35], and euhydration had a protective effect. Dehydration may lead to elevated blood pressure, functional burden, oxidative stress, and inflammatory responses in the kidney, ultimately causing proteinuria and kidney damage, through the activation of the renin–angiotensin–aldosterone system and release of vasopressin [15,16,36]. This finding underscores the vital role of maintaining adequate hydration in protecting kidney health.

Our study also revealed significant “dose-response”-like effects of sleep duration and hydration status on early renal impairment. Specifically, the combination of long sleep and dehydration was found to contribute more to kidney injury, while short sleep had a milder effect. These findings may be supported by the hypothesis that regulation of vasopressin release by the circadian rhythm mediates the association between sleep and hydration. During the late sleep period, vasopressin increases to help prevent dehydration [37,38,39] and may lead to renal impairment [36,40,41]. Furthermore, this study found adequate hydration had no significant protective effect on renal impairment among children with a long sleep duration, while a significant protective effect among children with short or optimal sleep duration. We hypothesize that children with long sleep even with euhydration bear prolonged sodium and protein excretion procedures, increased renal blood flow, and renal filtration burden, leading to an increased risk of transient tubular impairment. These findings can partially demonstrate the interaction of sleep and hydration on renal impairment as well.

The strengths of this study include the general healthy pediatric population, a large sample size, the use of four waves of urine assays to determine the longitudinal trends and associations, and the adjustment of key anthropometric and lifestyle covariates in the models. Multiple imputations were performed to complete and maximize the use of the collected data and reduce reporting bias. This study has several limitations. Given the scale of the epidemiological study, we did not consider other renal function indicators or biomarkers, while the use of two protein variables corresponded to different sites of kidney impairment. Given the limited capacity of the individual testing site for sample processing, urine indicators were tested via different machines across weekdays. However, these effects were minimized by longitudinal data applied with the random effect. The age span of the children was narrow (6–9 years old) as we conducted this child cohort among first-grade students, which limits the extrapolation of the results to adolescents. Meanwhile, the narrow age span before puberty and the one-year follow-up enabled us to make a robust and stable conclusion. The lifestyle questionnaires were completed with the assistance of parents, which may introduce potential recall bias. Given the cognitive capacity of children around 6 years old, it is standard practice to rely on parents to provide information, as young children may not fully understand the questions. Therefore, using guardians or parents as respondents is a routine method in such surveys. Additionally, the limitations inherent to observational studies should be acknowledged. In future studies, we may utilize animal models to investigate the specific mechanisms and pathways of injury through omics analysis. Furthermore, we intend to adopt a different approach in replication studies by incorporating other renal function indicators, hydration biomarkers, and a broader age range. This approach would help ensure a more comprehensive understanding of our findings.

## 5. Conclusions

In conclusion, children with suboptimal sleep (<9 h/d or ≥11 h/d) or with dehydration status were more likely to have transient renal impairment in the general pediatric population. These findings, that the combination of long sleep and dehydration contributes more to kidney injury, while short sleep duration also had a negative renal effect, though not as pronounced, reinforce the importance of healthy lifestyles, including promoting optimal sleep duration, maintaining adequate hydration, and monitoring of renal function even for healthy children.

## Figures and Tables

**Table 1:** Baseline characteristics categorized by sleep duration of 1914 children aged 6–8 in Beijing, China.

Characteristics	Short Sleep (*n* = 472)	Optimal Sleep (*n* = 1378)	Long Sleep (*n* = 64)	*p*
Boys [*n* (%)] ^a^	232 (49.2)	697 (50.6)	27 (42.2)	0.39
Age (year) ^b^	6.6 ± 0.3	6.6 ± 0.3	6.6 ± 0.3	0.69
Height z-score ^b^	0.65 ± 0.98	0.66 ± 0.96	0.80 ± 0.83	0.48
Weight z-score ^b^	0.76 ± 1.40	0.68 ± 1.42	0.73 ± 1.14	0.56
Body mass index z-score ^b^	0.50 ± 1.53	0.36 ± 1.55	0.33 ± 1.33	0.25
Systolic blood pressure (mmHg) ^b^	101 ± 9	101 ± 8	102 ± 9	0.61
Diastolic blood pressure (mmHg) ^b^	56 ± 6	56 ± 6	57 ± 6	0.40
Long screen time (≥2 h/d) ^a^	28 (5.9)	67 (4.9)	0 (0)	0.12
Insufficient physical activity (<1 h/d) ^a^	373 (79.0)	1036 (75.2)	42 (65.6)	0.037
Poor Mediterranean diet adherence ^a^	72 (15.3)	97 (7.0)	4 (6.3)	<0.001

^a^ Comparison by sleep duration using χ^2^ test or Fisher’s exact test. ^b^ Means and standard deviations (SDs) compared by sleep duration using analysis of variance.

**Table 2:** Longitudinal binary associations between sleep duration, hydration status, and renal tubular and glomerular impairment among children aged 6–9 years in Beijing, China.

Dependent Variable	Independent Variable	Model 1		Model 2		Model 3	
		cOR (95% CI)	*p*	aOR (95% CI)	*p*	aOR (95% CI)	*p*
Renal tubular impairment	Short sleep	1.04 (0.87, 1.16)	0.95	1.02 (0.88, 1.12)	0.79	1.01 (0.87, 1.17)	0.91
	Optimal sleep	1		1		1	
	Long sleep	1.84 (1.37, 2.48)	<0.001	1.89 (1.40, 2.55)	<0.001	1.88 (1.39, 2.54)	<0.001
Renal glomerular impairment	Short sleep	0.93 (0.72, 1.21)	0.61	0.94 (0.73, 1.23)	0.67	0.91 (0.70, 1.19)	0.50
	Optimal sleep	1		1		1	
	Long sleep	1.35 (0.79, 2.31)	0.28	1.27 (0.74, 2.19)	0.38	1.26 (0.73, 2.18)	0.40
Renal tubular impairment	Euhydration	1		1		1	
	Dehydration	2.19 (1.91, 2.50)	<0.001	2.10 (1.83, 2.40)	<0.001	2.10 (1.83, 2.40)	<0.001
Renal glomerular impairment	Euhydration	1		1		1	
	Dehydration	2.55 (1.98, 3.29)	<0.001	2.98 (2.29, 3.89)	<0.001	2.97 (2.27, 3.88)	<0.001

Model 1: unadjusted; model 2: adjusted for sex, age, and body mass index z-score; model 3: model 2 further adjusted for systolic blood pressure z-score, screen time, physical activity level, and Mediterranean diet adherence. cOR: crude odds ratio; aOR: adjusted odds ratio; CI: confidence interval. All models included one random effect: the weekday of the urine assay.

**Table 3:** Longitudinal multivariate associations between sleep duration, hydration status, and renal tubular and glomerular impairment among children aged 6–9 years in Beijing, China.

Dependent Variable	Independent Variable	Model 1		Model 2		Model 3	
		cOR (95% CI)	*p*	aOR (95% CI)	*p*	aOR (95% CI)	*p*
Renal tubular impairment	Short sleep	0.99 (0.85, 1.14)	0.84	1.01 (0.87, 1.16)	0.94	1.00 (0.86, 1.16)	0.98
	Optimal sleep	1		1		1	
	Long sleep	1.87 (1.39, 2.53)	<0.001	1.91 (1.41, 2.58)	<0.001	1.90 (1.40, 2.57)	<0.001
	Euhydration	1		1		1	
	Dehydration	2.19 (1.92, 2.50)	<0.001	2.10 (1.84, 2.40)	<0.001	2.10 (1.83, 2.40)	<0.001
Renal glomerular impairment	Short sleep	0.91 (0.70, 1.19)	0.50	0.92 (0.71, 1.21)	0.56	0.90 (0.68, 1.18)	0.45
	Optimal sleep	1		1		1	
	Long sleep	1.35 (0.79, 2.32)	0.28	1.24 (0.71, 2.17)	0.44	1.22 (0.69, 2.14)	0.49
	Euhydration	1		1		1	
	Dehydration	2.55 (1.98, 3.29)	<0.001	2.98 (2.29, 3.89)	<0.001	2.97 (2.27, 3.88)	<0.001

Model 1: unadjusted; model 2: adjusted for sex, age, and body mass index z-score; model 3: model 2 further adjusted for systolic blood pressure z-score, screen time, physical activity level, and Mediterranean diet adherence. cOR: crude odds ratio; aOR: adjusted odds ratio; CI: confidence interval. All models included one random effect: the weekday of the urine assay.

**Table 4:** Longitudinal binary associations between euhydration status and renal tubular and glomerular impairment by sleep duration among children aged 6–9 years in Beijing, China.

Group	Exposure	Renal Tubular Impairment		Renal Glomerular Impairment	
		OR (95% CI)	*p*	OR (95% CI)	*p*
Optimal sleep	Dehydration	1		1	
	Euhydration	0.43 (0.36, 0.50)	<0.001	0.33 (0.24, 0.46)	<0.001
Short sleep	Dehydration	1		1	
	Euhydration	0.62 (0.47, 0.81)	<0.001	0.31 (0.17, 0.55)	<0.001
Long sleep	Dehydration	1		1	
	Euhydration	0.61 (0.33, 1.13)	0.11	0.44 (0.09, 2.17)	0.32

Models adjusted for sex, age, and body mass index z-score; OR: odds ratio; CI: confidence interval.

## Data Availability

The data that support the findings of this study are not publicly available due to the vulnerable population being studied (children), but deidentified data are available from the corresponding author with a well-justified and documented request.
